# A phylogeny-based method in detecting the species-specialized genes in microbes and its application to a marine symbiont bacterium

**DOI:** 10.1128/mbio.03566-24

**Published:** 2025-10-29

**Authors:** Xiaolin Zhou, Weijie Li, Siya Xie, Kang Xia, Mei Xiao, Xiaojing Yang, Zhiyuan Li

**Affiliations:** 1 Center for Life Sciences, School of Life Sciences, Tsinghua University, Beijing, China; 2 Peking University Chengdu Academy for Advanced Interdisciplinary Biotechnologies, Chengdu, Sichuan, China; 3 Center for Life Science, Academy for Advanced Interdisciplinary Studies, Peking University, Beijing, China; 4 Center for Quantitative Biology, Academy for Advanced Interdisciplinary Studies, Peking University, Beijing, China; University of Tennessee at Knoxville, Knoxville, Tennessee, USA

**Keywords:** homologous recombination, species-special genes, phylogenetic analysis

## Abstract

Different microbes possess special traits that enable adaptation to their specific niches, often through specialized genes. Beyond orphan genes, few methods identify homologous genes with unusual functional regions. To address this, we investigated the extreme recombination frequency of the marine symbiont *Ca. E. kahalalidifaciens* and developed an unbiased computational approach to detect genes deviating from phylogenetic expectations. This method identified Rec family genes as key contributors to the exceptional recombination capacity in *Ca. E. kahalalidifaciens*. Heterologous expression in *Escherichia coli* demonstrated that these gene variants significantly enhance recombination efficiency. These efficient Rec variants offer potential for improving genetic manipulation tools, while our pipeline provides a versatile strategy for microbial genome mining.

**IMPORTANCE:**

Like how polar bears can survive extreme cold or how bats can navigate in darkness, different bacteria have special talents in surviving in their unique environments. However, finding the genes responsible for these special abilities is like looking for a needle in a haystack. We developed a new computer method for finding these special genes. We tested it on an amazing bacterium that lives inside marine algae and is exceptionally good at mixing and matching its DNA. We found the genes responsible for this ability and showed that when we put them into common laboratory bacteria, they gained this enhanced DNA-recombination ability too. This discovery is exciting because it gives scientists both a new way to find special genes in any bacteria and new tools that could make genetic engineering more efficient in laboratories around the world.

## INTRODUCTION

In the quest for survival, each microorganism exhibits its unique abilities to adapt to its niche (1). For example, *Deinococcus radiodurans* can withstand extreme levels of radiation through efficient DNA repair mechanisms (2), while *Pseudomonas syringae* produces ice-nucleating proteins that promote frost damage on plants to facilitate colonization (3). Another remarkable example comes from a marine symbiont, where the bacterium *Ca. E. kahalalidifaciens* lives intracellularly to marine alga *Bryopsis* sp. and provides chemical defense for its host (4). In *Ca. E. kahalalidifaciens*, 20 biosynthetic gene clusters exchange genetic material at an exceptionally high frequency—orders of magnitude higher than in other known species (5). Such intensive recombination constitutes a mode of “diversifying evolution,” generating chemical diversity in the host’s defensive compounds. Given the demands of such unique cellular functions, it is reasonable to expect that special genes underlie these processes. A key question remains: How shall we identify gene sets linked to each microbe’s special physiology, such as the extreme recombination frequency observed in *Ca. E. kahalalidifaciens*?

Identifying genes linked to such specialized functions is challenging. Orphan genes—without homologs in other lineages—represent typical type of species-specialized genes (6). However, their uniqueness often complicates functional studies, requiring laborious experimental validation (7). More importantly, many special traits rely on variants of shared homologous, not orphan genes (8). For example, *Sirt6* is involved in DNA damage repair and is widely distributed at the eukaryote level, while rodents with *Sirt6* variants divergent at the C-terminal exhibit substantial longevity (9). Another example is the housekeeping *rpoB* gene encoding bacterial RNA polymerase, where certain mutations help the strain to evade the suppression of antibiotics rifampin (10). In primates, systematic methods have been developed to investigate the human accelerated regions (HAR), revealing sequences that likely contribute to human-specific traits (11). However, in the vast genomes of microbes, there are presently relatively few comparable methods for identifying the special genes that give a bacterium its unique trait (12).

The search for such variants is complicated by microbial evolution. Frequent mutations generate extensive variability, making it hard to pinpoint gene variants linked to special traits (13, 14). Meanwhile, phylogenetic relationships shape the overall pattern of genetic variations, with recently diverged species sharing higher sequence similarity than distant relatives (15, 16). Thus, we can establish phylogeny-based expectations for sequence similarity within homologous groups. Variants that deviate significantly from these expectations are prime candidates for species-specialized genes, likely responsible for unique traits in specific organisms.

Here, we introduce a computational strategy to identify phylogeny deviation (PD) genes by detecting sequences that deviate from phylogenetic expectations. The PD score evaluates whether the evolutionary pattern of a protein in the target species (e.g., *Ca. E. kahalalidifaciens*) forms a distinct cluster separate from patterns in related species. Applying this method to *Ca. E. kahalalidifaciens* uncovered *recG*, *ruvA/B/C*, and *ligA* as candidates contributing to its exceptional recombination. Structural analysis and selection pressure assessments revealed key mutations in DNA-binding and interaction domains. Heterologous expression of these variants in *Escherichia coli* significantly enhanced recombination efficiency, offering potential tools for genetic engineering. This pipeline provides a computationally generalizable framework for identifying functional variants across microbial genomes.

## RESULTS

### Data overview and PD score calculation

**Fig 1:**
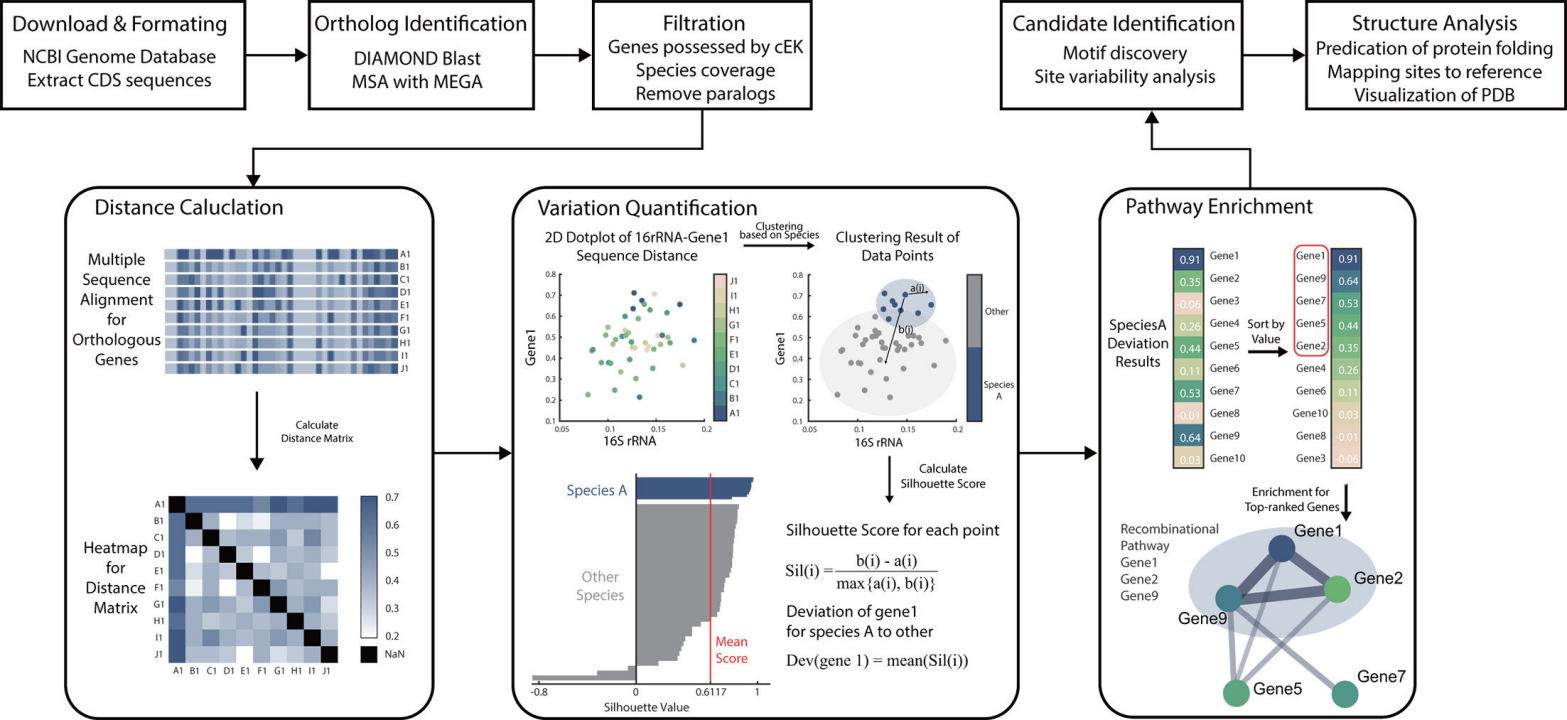
Method overview: the workflow for identifying phylogenetically deviated genes for a given species.

**Fig 2:**
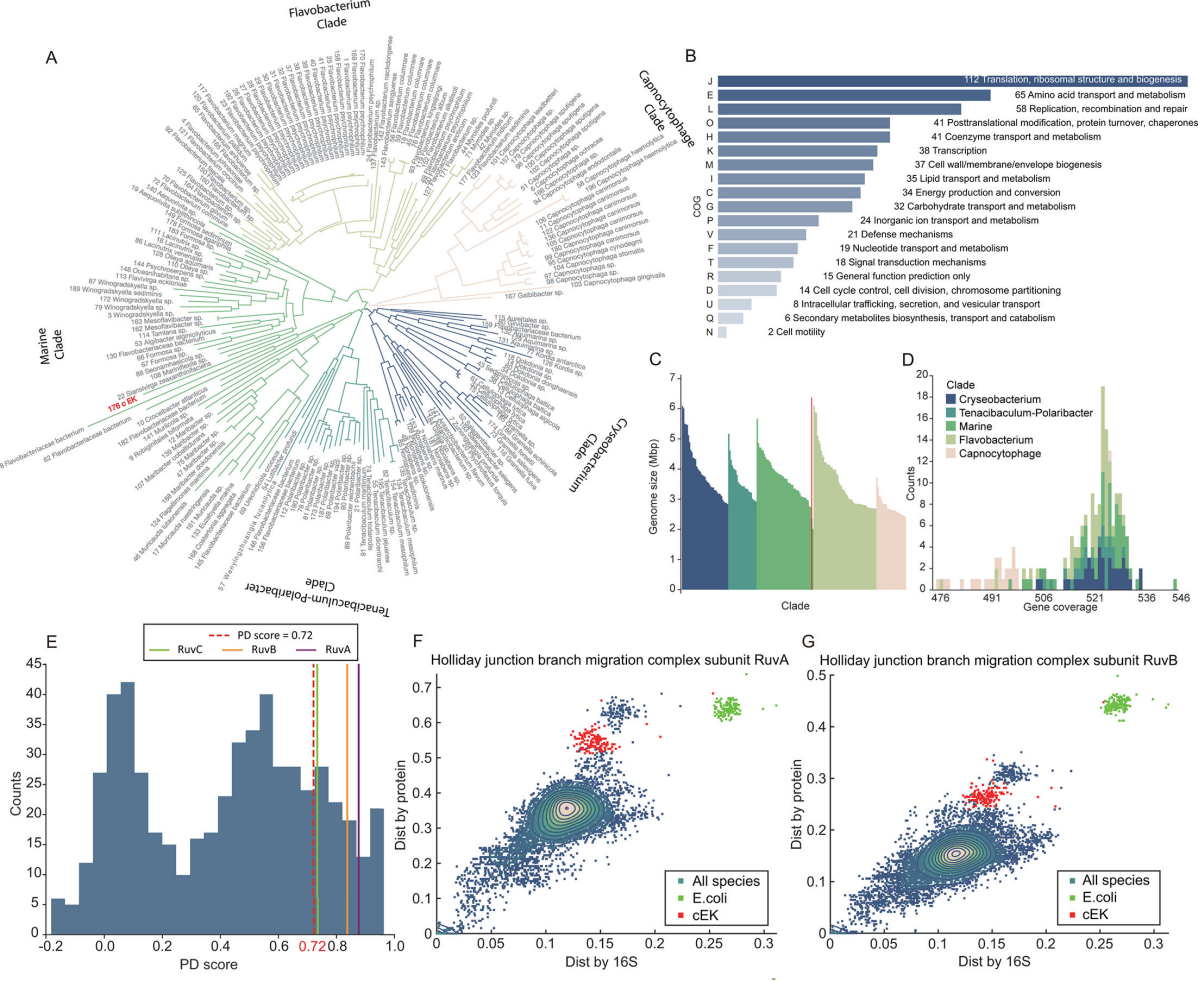
Overview of genomic data and PD score calculation. (**A**) Phylogenetic tree based on 16S rRNA sequences from 197 species, with *Ca. E. kahalalidifaciens* (cEK) highlighted in red. (**B**) Functional distribution of the 619 orthologous gene groups, categorized by COG classification. (**C**) Genome size distribution of the species shown in panel **A**, with colors corresponding to their clades. (**D**) Gene coverage across the 619 orthologous groups within each genome, colored by clade as in panel **A**. (**E**) PD score distribution for the orthologous gene groups in Ca. *E. kahalalidifaciens*. The red dashed line indicates the threshold (*z*-score = 1, selected to focus on genes in the upper tail of the PD distribution [approximately top 16%]). (**F and G**) Comparison between pairwise protein sequence distances and 16S rRNA distances for the Holliday junction branch migration complex subunits RuvA (**F**) and RuvB (**G**). Points in red represent cEK, green points represent *E. coli*, and blue points represent all other species.

We developed a computational framework to identify specialized genes that may underpin unique traits of given species (Fig. 1; see Methods and Materials for details). This approach was applied to a data set of 197 complete *Flavobacteriaceae* genomes, including *Ca. E. kahalalidifaciens* (cEK). Across these genomes, we identified 619 orthologous gene groups present in cEK (Fig. 2B). The genomes exhibit similar sizes and gene counts, as shown in Fig. 2C and D, ensuring a consistent data set for comparative analysis.

To quantify how much a gene’s protein-coding sequence deviates from expected phylogenetic patterns, we developed the phylogeny deviation score (PD score). This metric, ranging from −1 to 1, measures the extent of deviation, with higher values indicating greater divergence from the typical phylogenetic relationship (see Materials and Methods for details).

We calculated PD scores for all 619 orthologous gene groups in cEK, yielding a distribution centered around 0.5 (Fig. 2E). To illustrate the concept and application of PD scores, we examined two key components of the Holliday junction branch migration complex: RuvA and RuvB. Figure 2F and G highlight the relationship between 16S rRNA-based phylogenetic distances (*x*-axis) and the corresponding distances in protein-coding sequences (*y*-axis). For most species, the distances align closely along the diagonal, reflecting the expected evolutionary relationship. However, the red points representing cEK form distinct clusters, deviating significantly from the general trend. These outliers suggest that RuvA and RuvB have undergone sequence modifications specific to cEK, potentially contributing to its unique recombination abilities.

### Unbiased search revealed that both metabolic pathways and homologous recombination processes are highly specialized in *Ca. E. kahalalidifaciens*

We selected the top 100 genes for enrichment analysis, focusing on Cellular Components and Biological Processes (see Materials and Methods for details). The Cellular Component analysis (Table 1; Table S1) identified the Holliday junction resolvase complex as highly enriched with significant confidence. In parallel, the Biological Process analysis (Table 2; Table S2) showed a strong enrichment of nucleotide metabolism and translation regulation pathways, reflecting the role of cEK as an intracellular symbiont. Additionally, the recombinational repair process emerged with a high enrichment strength and a low false discovery rate (FDR), suggesting it plays a crucial role in the bacterium’s physiology.

**TABLE 1:** Cellular component enrichment with the false discovery rate lower than 0.01 and the strength greater than 0.75

GO-term	Description	Count in network	Strength	False discovery rate
GO: 0048476	Holiday junction resolvase complex	3 of 6	1.35	0.0072
GO: 0022625	Cytosolic large ribosomal subunit	6 of 32	0.92	0.0021

**TABLE 2:** Biological process enrichment with the false discovery rate lower than 0.02 and the strength greater than 1

GO term	Description	Count in network	Strength	False discovery rate
GO: 0006432	Phenylalanyl-tRNA aminoacylation	2 of 2	1.65	0.0158
GO: 0006438	Valyl-tRNA aminoacylation	2 of 2	1.65	0.0158
GO: 0006450	Regulation of translational fidelity	5 of 11	1.3	0.00016
GO: 0106074	Aminoacyl-tRNA metabolism involved in translational fidelity	4 of 9	1.29	0.0012
GO: 0006418	tRNA aminoacylation for protein translation	11 of 26	1.27	6.09E−09
GO: 0006189	“*De novo*” IMP biosynthetic process	5 of 12	1.27	0.00022
GO: 0046040	IMP metabolic process	6 of 6	1.22	5.98E−05
GO: 0000725	Recombinational repair	3 of 10	1.12	0.0145
GO: 0018205	Peptidyl-lysine modification	3 of 11	1.08	0.0176

Homologous recombination is an energy-consuming and high-fidelity process that demands cellular resources (17, 18). In most microorganisms, the homologous recombination pathways are conserved (18, 19). For the model organism *E. coli*, two general pathways of homologous recombination have been identified. One pathway operates at double-stranded DNA molecules using RecBCD, followed by RecA and either the RuvABC complex or RecG (20). Another pathway utilizes RecF, RecO, and RecR to deal with the single-strand break, followed by RecA, then either RuvABC or RecG. In both pathways, RecA, RuvABC complex, RecG, and lastly DNA ligase (to ligate DNA) are needed (21).

**Fig 3:**
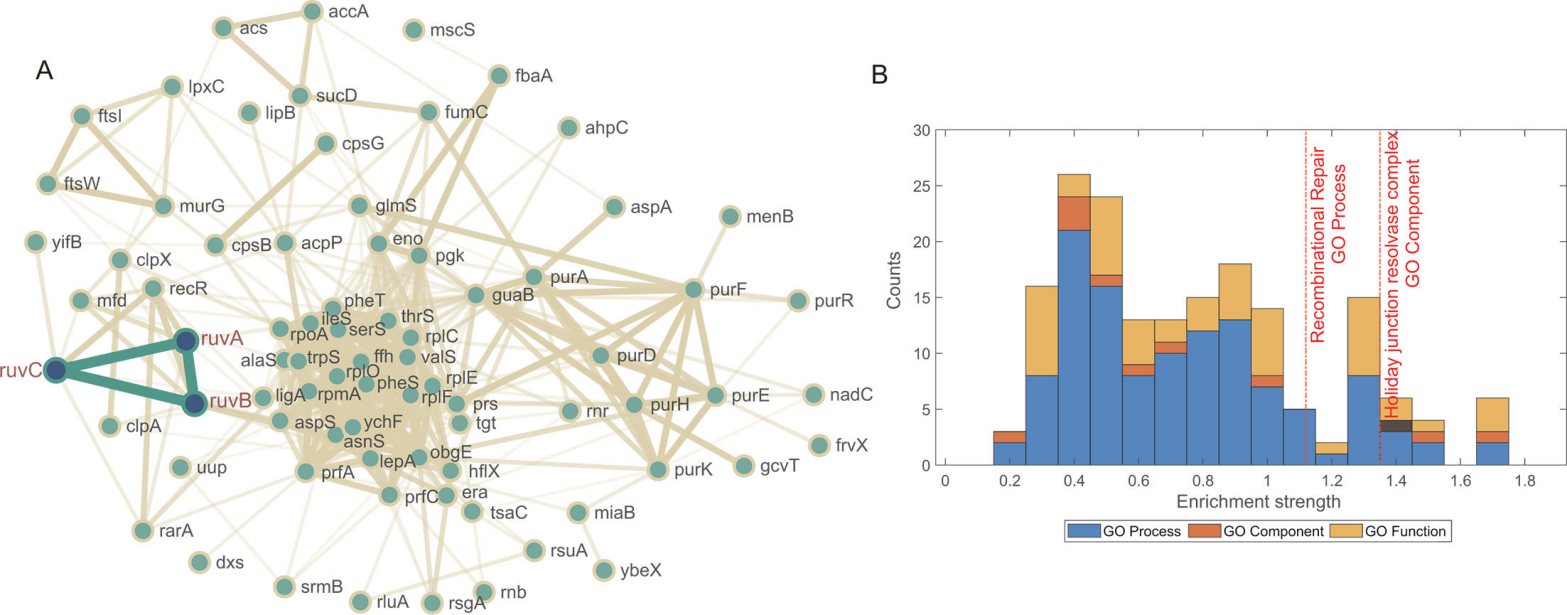
Gene ontology enrichment analysis for *Ca. E. kahalalidifaciens*-special genes. (**A**) Gene interaction network generated by STRING, with ruvA, ruvB, and ruvC highlighted in green. (**B**) Statistics of gene enrichment. The enrichment strength is log10(observed/expected).

We further analyzed recombination-related genes with PD scores above 0.72 (*z*-score ≥ 1) to identify candidates specific to cEK (Fig. 3A). The analysis pinpointed several key members of the Holliday junction resolvase complex: RuvA, RuvB, and RuvC, with PD scores of 0.88, 0.84, and 0.73, respectively. Additionally, we have also calculated Calinski-Harabasz index (CHI) and Davies-Bouldin index (DBI) scores for each gene and detected all these three genes in top and tail 100 genes, respectively (Tables S3 and S4). LigA, a NAD-dependent DNA ligase, also showed a high PD score of 0.77, which were also detected in CHI (ranking top 101 from largest to smallest) and DBI scores (ranking bottom 98 from largest to smallest). The distribution of enrichment strengths (Fig. 3B) confirms the high significance of recombinational repair, with the Holliday junction resolvase complex marked by strong enrichment in the Cellular Component category. In contrast, members of the Rec family displayed lower scores, with RecG at 0.71, RecA at 0.43, and RecQ at 0.01, indicating they may play less specialized roles in this species. Interestingly, the replication-associated recombination protein RarA, with a PD score of 0.80, also emerged as a candidate although its precise role in DNA repair remains under investigation (22). These results suggest that the recombination machinery of cEK may have undergone specific adaptations, possibly contributing to its enhanced recombination capacity.

### Sequence and structural analysis of *Ca. E. kahalalidifaciens*-specialized recombination-related Genes

Given that our unbiased PD score analysis revealed several recombination-related genes to be highly specialized in *Ca. E. kahalalidifaciens*, we conducted a detailed investigation of six key genes: *ruvA*, *ruvB*, *ruvC*, *recA*, *recG*, and *ligA*. The d*N*/d*S* ratio, comparing nonsynonymous to synonymous substitution rates, was used here as a correlative tool to explore potential links between these genes’ specialized functions and their structural basis, with values >1 suggesting positive selection. Despite that it does not provide direct evidence of adaptive evolution, such analysis provides clues for further functional assays and structural validations in future work.

**Fig 4:**
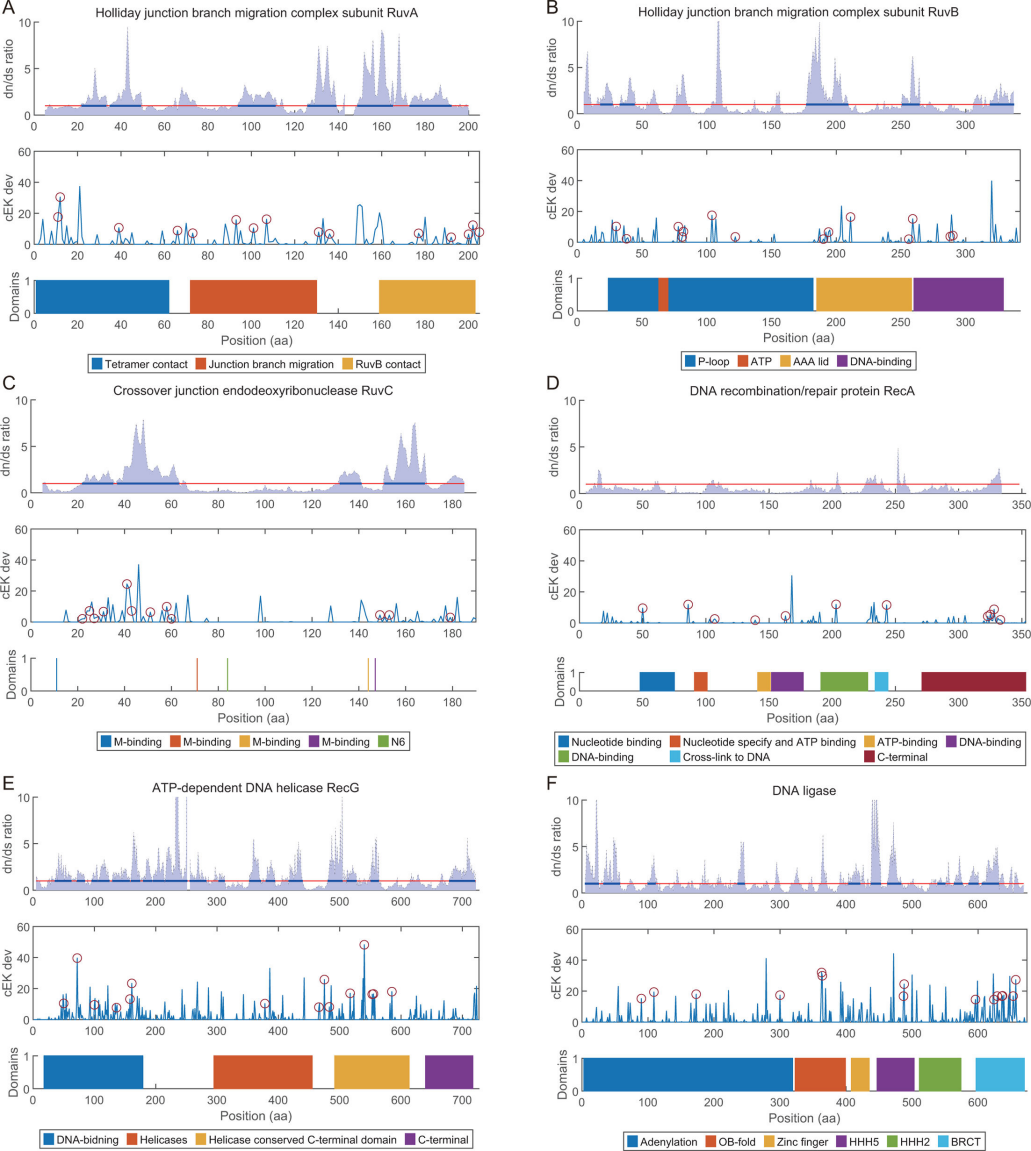
Sequence analysis of *Ca. E. kahalalidifaciens*-specialized recombination-related proteins. (**A–F**) Analysis of six recombination-related proteins: RuvA, RuvB, RuvC, RecA, RecG, and LigA. The top panel in each subplot displays the non-synonymous/synonymous mutation ratio (d*N*/d*S*) per codon site for *Ca. E. kahalalidifaciens* (cEK) compared to 196 other *Flavobacteriaceae* species. The red line indicates the threshold of d*N*/d*S* = 1, with regions above this threshold for at least 10 consecutive sites highlighted in blue. The middle panel shows the amino acid distance per site between cEK and other species, with red circles marking the 15 sites with the highest deviation that also differ from the *E. coli* sequence. The bottom panel provides functional site annotations and domain structures for each protein.

**Fig 5:**
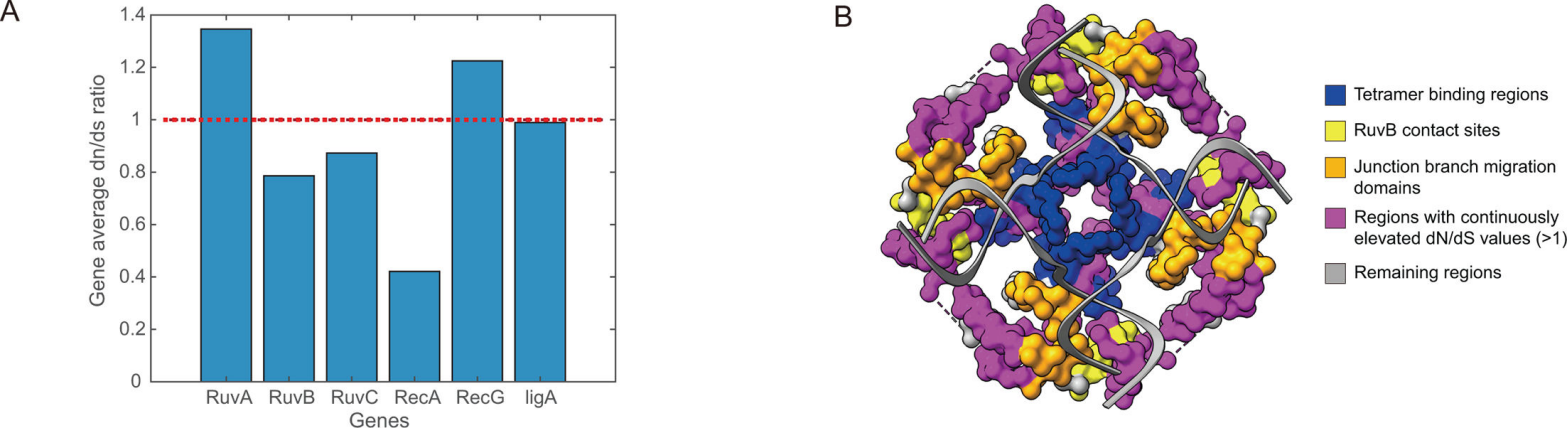
Selection pressure and structural analysis of *Ca. E. kahalalidifaciens*-specialized recombination-related proteins. (**A**) Average d*N*/d*S* ratios for the six proteins in cEK compared to other *Flavobacteriaceae* species. The red dashed line marks the neutral evolution threshold (d*N*/d*S* = 1). (**B**) Structural model of RuvA bound to a Holliday junction. The RuvA tetramer is colored according to the functional domains: blue indicates tetramer binding regions, orange denotes junction branch migration domains, yellow highlights RuvB contact sites, and the remaining regions are shown in gray. Regions with continuously elevated d*N*/d*S* values (>1) are highlighted in purple, indicating potential adaptive changes.

We calculated their d*N*/d*S* ratios, comparing cEK to 196 other *Flavobacteriaceae* species. As shown in Fig. 4G, *ruvA* exhibited the highest average d*N*/d*S* ratio (1.35), followed by *recG* (1.22), while *recA* showed the lowest ratio (Fig. 5A). We further examined the d*N*/d*S* values and deviation scores across each protein’s sequence to identify regions subject to positive selection (Fig. 4A through F). *RecA*, which initiates recombination by facilitating single-strand DNA invasion (23), displayed low PD scores (0.43) and did not show signs of significant positive selection in cEK (Fig. 4D). This suggests that *recA* has not diverged considerably from its phylogenetic relatives in cEK.

The Holliday junction, formed after RecA-mediated strand invasion, is processed by a complex consisting of a RuvA tetramer, two RuvB hexameric rings, and a RuvC dimer. Together, these proteins facilitate junction migration and resolution (24). Within this complex, RuvA binds to DNA and senses structure, RuvB provides ATP-driven energy for migration, and RuvC acts as an endonuclease to resolve the junction (25).

Our analysis revealed that domains involved in protein-protein interactions within the RuvA/B/C complex are under strong positive selection in cEK (Fig. 4A through C). For RuvA in *Ca. E. kahalalidifaciens*, selective pressure was concentrated in three domains responsible for tetramer contact, junction migration, and RuvB interaction (Fig. 4A and 5B). In other species, these three regions have been reported to be highly conserved (26). For RuvB, its N and M domains are responsible for the RuvA-RuvB interaction and RuvB hexamer assembly, while the C domain is responsible for DNA binding (27). In our result, high positive selection pressure was observed in the RuvA-RuvB and RuvB-RuvB interaction regions (Fig. 4B). The RuvC protein can both bind to DNA and, with the existence of Mg2+, catalyze sequence-specific cleavage (26). Multiple active sites have been identified for catalysis sequence specificity, yet few studies have divided the function of RuvC into different domains as RuvA/B does. In our observation, two regions in RuvC (residues 15–28 and residues 30–55) were detected with strong positive selection in *Ca. E. kahalalidifaciens* (Fig. 4C), indicating potential functional divergence.

Other than the RuvA/B/C complex, RecG, a DNA-dependent ATPase, is also capable of contacting with Holliday junction directly to drive branch migration (28). RecG comprises multiple functional domains: the N-terminal wedge domain interacting with the DNA junction, the helicases and the helicase conserved C-terminal domain, and the C-terminal domain that forms a hook with the first domain to wrap around the extended alpha-helix (29). In our observation, all these functional domains experience high positive selection in *Ca. E. kahalalidifaciens* (Fig. 4E). Of note, most *Ca. E. kahalalidifaciens*-specific mutations concentrate in the helix-forming domains known to be conserved in most microorganisms (30).

LigA, the NAD-dependent DNA ligase that catalyzes the formation of phosphodiester linkages between 5′-phosphoryl and 3′-hydroxyl groups in double-stranded DNA, functions at the last step of recombination in ligating the DNA strand (31, 32). More importantly, it has been suggested to function in other non-homologous DNA repair pathways, including the non-homologous end-joining (NHEJ) (33) and the alternative end-joining (A-EJ) pathway (34), and offers potentials for efficient CRISPR-Cas9-assisted genetic engineering in microbes (35). In *Ca. E. kahalalidifaciens*, the functional domains of LigA consistently exhibit a high dN/dS ratio (Fig. 4F), particularly at the BRCA1 C-terminal, the integral signaling module in the DNA damage response (36). As LigA is involved in multiple DNA damage response pathways, its *Ca. E. kahalalidifaciens*-specific mutations may bias it toward homologous recombination repair.

In summary, these sequence and structural analyses reveal targeted positive selection in functional domains of cEK’s recombination-related genes, offering correlative clues to their specialized roles in enhancing recombination efficiency. Although d*N*/d*S* and modeling do not provide direct causal evidence, they bridge evolutionary deviations to potential structural adaptations, paving the way for future mechanistic studies via site-directed mutagenesis and functional assays to confirm these links.

### *Ca. E. kahalalidifaciens* proteins significantly enhance homologous recombination in *E. coli*

Our bioinformatic analysis identified several *Ca. E. kahalalidifaciens* (cEK) proteins with high potential to enhance homologous recombination. Based on these findings, we hypothesized that the cEK recombination system has evolved unique adaptations to increase recombination efficiency, and we proceeded to validate this experimentally.

**Fig 6:**
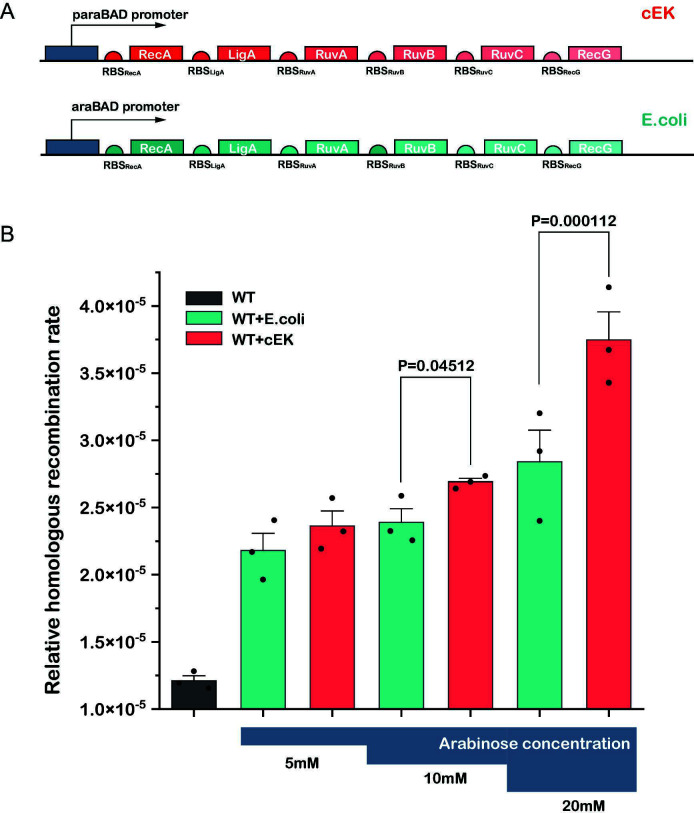
*Ca. E. kahalalidifaciens* proteins significantly enhance homologous recombination in *E. coli.* (**A**) Schematic representation of plasmid constructs used to express homologous recombination systems. The upper panel illustrates the homologous recombination system derived from *Ca. E. kahalalidifaciens* (cEK), while the lower panel shows the native *E. coli* system, both under the control of the arabinose-inducible paraBAD promoter. (**B**) Comparison of homologous recombination rates across three experimental groups: wild-type *E. coli* (gray), wild-type *E. coli* expressing the native *E. coli* recombination system (teal), and wild-type *E. coli* expressing the cEK recombination system (red). Recombinational efficiency was measured at three arabinose concentrations (5 mM, 10 mM, and 20 mM). *P* values indicating statistical significance are shown.

To test whether the cEK recombination proteins enhance homologous recombination in *E. coli*, we introduced the cEK recombination system into wild-type *E. coli* MG1655 and measured recombination rates. For comparison, we also introduced an additional copy of the native *E. coli* recombination system as a control. To optimize expression in *E. coli*, we adjusted codon usage and ribosome binding sites (RBS) for each cEK gene. All six genes were organized in a single operon under the control of an inducible promoter, arranged according to their endogenous expression levels in *E. coli* to facilitate efficient protein interaction (Fig. 6A; see Materials and Methods for details). By using gradient arabinose induction, we ensured that any observed changes in recombination rates were due to the introduced cEK proteins.

The results showed that the expression of the cEK recombination system significantly increased homologous recombination rates in *E. coli* compared to the native *E. coli* system, with the effect becoming more pronounced at higher induction levels (Fig. 6B).

## DISCUSSION

In this study, we identified and experimentally validated the role of *Ca. E. kahalalidifaciens* (cEK) proteins in enhancing homologous recombination. Using a PD score-based bioinformatic approach, we pinpointed recombination-related genes with high potential for species-specialized adaptation. Experimental validation in *E. coli* showed that the cEK recombination system significantly improved recombination rates, supporting the hypothesis that cEK’s recombination machinery has evolved for enhanced efficiency. These findings reveal unique aspects of the cEK homologous recombination pathway and suggest possible applications in genetic engineering.

Our analysis highlighted substantial positive selection in key recombination proteins RuvA, RuvB, RuvC, RecG, and LigA. Recombination has been a key factor in genetic engineering (37). Despite that most engineering efforts in optimizing recombination efficiency have been put on RecA (38), as well as its eukaryote ortholog Rad51 (39), the conserved nature of RecA in *Flavobacteriaceae* suggests that it may maintain an essential role in initiating homologous recombination, which may be more challenging to optimize further without compromising its essential functions. In contrast, the RuvA/B/C complex in cEK appears to have undergone substantial adaptive modifications, likely enabling fine-tuning downstream processes like branch migration and junction resolution. Our finding implies that engineering efforts in recombination could benefit from focusing on the Ruv complex, as they show greater evolutionary flexibility. Our d*N*/d*S* analysis detected ratios >1 in functional domains of these proteins, providing correlative evidence for positive selection potentially driving enhanced recombination through evolutionary pressure favoring advantageous amino acid changes. Structural analyses and domain comparisons identified key mutations in DNA-binding and protein-interaction sites that may underlie these adaptations. Although our approach represents an initial step in linking sequence variation to structural and functional specialization, rigorous validation through site-directed mutagenesis and biophysical assays will be essential to definitively establish causality.

Beyond our specific findings, this work underscores the value of the PD score as a powerful, expandable, and intuitive method for identifying species-specialized genes. It does not constrain the specific function the gene performs, just detecting anything that deviates from the phylogenetic expectation, allowing researchers to isolate candidates for specialized traits in diverse microbial genomes. The simplicity of the method also makes it accessible for broad applications across various fields, from evolutionary biology to synthetic biology, enabling the discovery of lineage- specialized adaptations without prior knowledge of gene function (40).

While our study provides important insights, several limitations remain. The PD score relies on full sequence comparisons, which may miss genes with functionally significant but subtle sequence changes. Additionally, our analysis focuses on coding sequences; non-coding regulatory elements could also play a role in the evolution of specialized traits and warrant further investigation (41). Furthermore, our application to cEK only serves as a proof-of-concept case study, demonstrating the pipeline’s efficacy in one microbial system. While the method is computationally generalizable to other species for identifying specialized genes, future work should extend experimental validations across a broader range of organisms to confirm functional outcomes and refine predictive accuracy. Future improvements could incorporate structural analysis and experimental validation across a broader range of organisms to refine the PD score’s predictive power. By expanding these capabilities, the PD score could become a key tool for uncovering specialized genetic adaptations across diverse lineages.

## MATERIALS AND METHODS

### Data collection and reformatting

Complete genomes from 222 species of the *Flavobacteriaceae* family were downloaded from the NCBI Genome database in GenBank format. Twenty-six genomes were excluded due to the absence of 16S rRNA annotations, leaving a total of 196 species for analysis. The genome of *Ca. E. kahalalidifaciens* was obtained from the work of Zan et al. (4).

Gene data from each genome were parsed into annotations, nucleotide, and amino acid sequences, with genes lacking clear annotations (e.g., pseudogenes, ambiguous regions, non-methionine starts, or mismatched translations) excluded from further analysis. 16S rRNA sequences were extracted to assess phylogenetic relationships, and when multiple sequences were present in a genome, the one with the least mean distance to others was chosen as the representative sequence.

### Orthologous group identification and gene filtering

*Escherichia coli* K-12 is a classic model organism for bacterial genetics and molecular biology. Its extensive characterization makes it an excellent reference, with over two-thirds of its genes having experimentally confirmed functions (42). We chose to use it as a reference with 4,242 protein-coding genes. This is especially helpful for annotating uncultured bacteria, whose gene functions are predicted computationally by comparing them to experimentally characterized genes from model organisms like *E. coli* K-12 (43). Homology was inferred using DIAMOND v2.0.5.143: a protein database was built with the *E. coli* proteome (diamond makedb --in ecoli_protein.fa --db ecoli_protein), and sequences from 197 species (including *Ca*. *E*. *kahalalidifaciens*) were aligned (diamond blastp --db ecoli_protein -q all_flavo.fa -o protein_matches.txt). We applied an *E*-value cutoff of 1e−5 and a minimum sequence identity of 30%, standard thresholds in comparative genomics to balance sensitivity and specificity. For multiple sequences per species in an orthologous group, the most similar to the *E. coli* reference was selected. Of 619 orthologous groups, those from *Ca. E. kahalalidifaciens* were named and ID-matched to *E. coli* for further analysis.

### Multiple sequence alignment and calculation of sequence distances

Multiple sequence alignments for the 619 orthologous groups and 16S rRNA sequences were performed using MUSCLE in MEGA-CC v.10.1.8 with the neighbor-joining method. Pairwise distance matrices were computed under the Jones-Taylor-Thornton (JTT) model with a Gamma distribution (parameter = 1).

The amino acid sequence distance between species a and b in the g-th orthologous group is denoted as $O_{g}(a,b)$. The 16S rRNA nucleotide sequence distance between species a and species b is denoted as Phy(a,b), representing the phylogenetic difference. Assuming there are N species, then both matrices are N×N in size. To avoid bias induced by differences in the evolutionary rates between ortholog groups, both distances were centralized and scaled to the unit variance. Specifically, for each distance matrix representing pairwise evolutionary distances among species for a given ortholog group, the following steps were performed:

- Centralization: the mean of the distance values within each matrix was subtracted from each distance value. This centers the data around zero, removing any offset that might arise from varying evolutionary rates across ortholog groups.
- Scaling to unit variance: the centralized distances were then divided by the standard deviation of the original distance values, transforming the data to have unit variance. This standardizes the relative spread of distances, mitigating the impact of innate differences in evolutionary rates due to varied selective pressures.

This standardization process was applied consistently to both the distance matrices of *Ca. E. kahalalidifaciens* (cEK) ortholog groups and the pairwise distances derived from the 16S rRNA phylogenetic tree.

### Scoring orthologous genes in given species by deviations from phylogeny

To identify special genes, for each orthologous group g, we compared its amino acid sequence distances $O_{g}$ against the 16S distances Phy between all pairs of species. The protein sequence divergence is represented by $O_{g}$, whereas the phylogenetic distance is represented by Phy. Using Phy as the *x*-value and $O_{g}$ as the *y*-value, we established a “distance space” in which each data point is a two-element vector:


$$\vec{v_{g}}\left(a,b\right)=\left[Phy\left(a,b\right),\;O_{g}\left(a,b\right)\right],$$

between species a and species b (total species number N). In this distance space, protein sequence divergence $O_{g}$ tends to scale with phylogenetic distance Phy by default. We defined a phylogeny deviation score (PD score) based on thesilhouette index (44) to quantify deviation from this default trend, as described below:

A given species c divides all data points ${\vec{v}}_{g}$ in the distance space into two clusters: the first cluster $C_{c}$ containing points that are related to species c ($\vec{v_{g}}(c,k)\in C_{c}$, for k=1…N), and the second cluster $C_{other}$containing points that are unrelated to species c ($\vec{v_{g}}(a,b)\in C_{other}$,a≠c∧b≠c). The silhouette coefficient quantifies how well a data point in one cluster separates from points in other clusters (44). To calculate the silhouette coefficient, we first computed the Euclidean distance $d_{g}(i,j)$ between each pair i,j of $\vec{v}$ in the distance space:


$$d_{g}\left(i,j\right)=\sqrt[{2}]{{\left(Phy\left(a\left(i\right),b\left(i\right)\right)-Phy\left(a\left(j\right),b\left(j\right)\right)\right)}^{2}+{\left(O_{g}\left(a\left(i\right),b\left(i\right)\right)-O_{g}\left(a\left(j\right),b\left(j\right)\right)\right)}^{2}}.$$

Then, for the i-th point in the first cluster $C_{c}$, let $h_{c,g}(i)$ represent the mean Euclidean distance between this point and all other points in $C_{c}$:


$$h_{c,g}\left(i\right)=\frac{1}{\left|C_{c}\right|-1}\sum_{j\in C_{c},i\neq j}d_{g}(i,j),$$

where $\left|C_{c}\right|$ is the size of number of points belonging to the first cluster.

And we let f(i) represent the mean distance of the i-th point in the first cluster $C_{c}$ to all points in the second cluster $C_{other}$:


$$f_{c,g}\left(i\right)=\frac{1}{\left|C_{other}\right|}\sum_{j\in C_{other},}d_{g}(i,j),$$

where $\left|C_{other}\right|$ is the size of number of points belonging to the second cluster.

The silhouette coefficient of the i-th point in the first cluster $C_{1}$, by definition (44), is calculated as:


$$s_{c,g}\left(i\right)=\frac{f_{c,g}\left(i\right)-h_{c,g}\left(i\right)}{\max\left(f_{c,g}\left(i\right),h_{c,g}\left(i\right)\right)},$$

with $s_{c,g}\left(i\right)$ ranges from −1 to 1. A high value indicates that the i-th point is well matched to the first cluster and clearly distinguishable from the second cluster. Therefore, the averaged silhouette coefficient for all points in the first cluster $C_{c}$ reflects how much the protein-phylogeny relationships for species c deviate from these in all other species. We defined it as the SSscore(c,g) for species c in the ortholog gene g:


$$SSscore\left(c,g\right)=\frac{1}{\left|C_{c}\right|}\sum_{i\in C_{c},}s_{c,g}\left(i\right).$$

This score indicates how special this variant in the orthologous group g is for the species c, compared with the default protein-phylogeny relationship.

### Pathway enrichment analysis

As proof of concept, we ranked the PD scores for all orthologous groups in *Ca. E. kahalalidifaciens* from highest to lowest. The top 100-ranked genes, identified by their *E. coli* names, were subjected to pathway enrichment analysis using the STRING online server.

### Motifs and structural analysis

For the gene of interest, we employed MEME Suite to detect sequence motifs within its orthologous group. PDB files for the 3D structure of *E. coli* RuvA tetramer were downloaded from the NCBI PDB database, and the 3D structure of RuvA in *Ca. E. kahalalidifaciens* was predicted using the RaptorX online server.

### Strains and culture medium

All cloning and testing experiments were conducted using *Escherichia coli* K-12 sub strain MG1655. Cells were grown in LB medium (10  g/L tryptone, 5  g/L yeast extract, and 10  g/L NaCl). For agar plates, 15  g/L agar was added to the medium. To select and maintain plasmids, antibiotics were used at concentrations of either 100  µg/mL ampicillin or 50  µg/mL kanamycin. All chemicals used in this study were purchased from Sigma–Aldrich unless otherwise stated.

### Gene codon and expression optimization

To optimize the simultaneous overexpression of recombination-related proteins, including RecA, LigA, RuvA, RuvB, RuvC, and RecG, in *E. coli* MG1655, we first performed codon optimization tailored to this specific expression host. The ribosome binding sites (RBS) for each protein were subsequently calculated based on the optimized codon sequences (Table 3) using the *De Novo* DNA platform (https://www.denovodna.com/). The genes were then arranged in order of their predicted endogenous expression levels in *E. coli*, from low to high, according to data from EcoCyc (http://ecocyc.org). These sequences were synthesized by GeneWiz and BGI for further experimental analysis.

**TABLE 3:** The calculated optimal RBS used in this study

Protein	Optimal RBS (5′−3′)
RecA-*Ca. E. kahalalidifaciens*	cggcagtctgcactgcttagagtcgagttaataaggaggtatttt
LigA-*Ca. E. kahalalidifaciens*	caaacggtatctgaccgtttatacgattaagaagataaggaggtattta
RuvA-Ca. *E. kahalalidifaciens*	ttggacgtcggaacatctaccgaattaagaaataaggaggttatat
*RuvB-Ca. E. kahalalidifaciens*	gcgggtcctactggacccgaaacatatcaacgcattaaggaggttatatt
RuvC-*Ca. E. kahalalidifaciens*	ccatcgatcgttgccttggcaacgcttcactcaattaaggaggtatttta
RecG-*Ca. E. kahalalidifaciens*	ggtcccgctcgataagcgagcatatcatttaaggaggtaattt
RecA-mg1655	tggtccctgagagggggtaatttaaactgcgcgcactaaggaggtatat
LigA-mg1655	acgcacatcgtaatagtaaggaggttttcg
RuvA-mg1655	tgaaaactcctgcaatactgcagggacagataggtaaggaggtatttg
RuvB-mg1655	gcgcgacgctacgcgttgcgatatagaacgaatcactaaggaggttttga
RuvC-mg1655	ctcacgacacgcggctctcgccgcaccagacaataaggaggtatttt
RecG-mg1655	tgagttgaattttcgcgagataaggaggagtttat

### Plasmid construction

To enable controlled overexpression of the target proteins, we utilized the L-arabinose-inducible araBAD promoter. Given the potential metabolic burden associated with overexpression, we opted for a medium-copy vector, using the p15A origin (~15–30 copies per cell). A kanamycin resistance marker was selected for the expression vector to ensure compatibility with the Ampicillin resistance in the homologous recombination assay kit. A complete list of primers used is provided in Table 4.

**TABLE 4:** The primers used in this experiment

Primers	Sequence (5′−3′)
MCS F	ggctacggtctcgaataatggatccgaattcgagctccgtcgacaagcttgcggccgcactcgagtgataagccagcatcaataaaacgaaagg
MCS R	ggctacggtctcatatttctagaggactagtattatacctaggactgagctagctgtcaattggtaacgaatcagacaattgacgg
Kana F	ggctacggtctcgttagaaaaactcatcgagcatcaaatgaaactg
Kana R	ggctacggtctcagaagatcctttgatcttttctacggggt
P15J23 Kana F	ggctacggtctcgcttcctgtcagaccaagtttactcatatatacttt
P15J23 Kana R	ggctacggtctcactaaagagtttgtagaaacgcaaaaag
BAD promoter F	gttaccaattatgacaacttgacggctacatcatt
BAD promoter R	tctagaggatggagaaacagtagagagttgcgata
BAD vector F	gtttctccatcctctagaaataatggatccgaattcgagc
BAD vector R	gttgtcataattggtaacgaatcagacaattgacggc

We amplified a linear backbone (P15J23A) from the P4218 plasmid (kindly provided by ZhiSun) using MCS F and MCS R primers, which included the p15A origin, Ampicillin resistance, J23119 promoter, MCS, and a strong terminator. The linearized backbone was circularized using the Golden Gate reaction. The Kanamycin resistance gene was amplified from pET-28a using Kana F and Kana R primers, digested with DpnI, purified, and cloned with the backbone via Golden Gate assembly to create p15J23K. The araBAD promoter was amplified from the pCas plasmid using BAD promoter F and BAD promoter R primers. This fragment was assembled with a linearized vector fragment, derived from p15J23K using BAD vector F and BAD vector R primers, via Gibson assembly, yielding the P15BAD vector. Overexpression fragments for recombinase proteins from *Ca. E. kahalalidifaciens* and *E. coli* MG1655 were inserted into P15BAD using BamHI and HindIII digestion, producing p15BAD-*Ca. E. kahalalidifaciens* and p15BAD-mg1655, with construction confirmed by sequencing.

### Recombination system introduction

For the homologous recombination assay, we utilized the Homologous Recombination Assay Kit (Norgen Biotek, Product #35600, Canada). The plasmids p15BAD-*Ca. E. kahalalidifaciens* and p15BAD-mg1655 were first electroporated into *E. coli* MG1655 cells. Fresh competent cells carrying the overexpression plasmids were prepared the following day through repeated procedures. L-arabinose was added to a final concentration of 10 mM 1 h before cell harvesting to induce the expression of recombination-related proteins.

Afterward, 25 ng of each DL-1 and DL-2 plasmids (from the assay kit) was co-transformed into the prepared competent cells. The transformation was performed at a 1:100 dilution into a liquid medium containing both Kanamycin and Ampicillin, followed by overnight culture. All transformations were done in triplicate, with MG1655 cells without the overexpression plasmid serving as the negative control.

### Quantitative analysis of recombination

Plasmid DNA was extracted from the cultures and adjusted to 20 ng/µL for further qPCR analysis. This analysis aimed to quantify the recombination rate by amplifying plasmids with both universal and assay-specific primers. Universal primers targeted all plasmids (DL-1, DL-2, and the recombined positive plasmid), while assay-specific primers amplified only the recombined plasmid. Recombination rates were calculated based on CT values obtained during qPCR with the following formula:


$$homologous\;recombination\;rate=\frac{2^{CT_{universal}}}{2^{CT_{assay}}}$$

The relative recombination efficiency can then be determined based on the number of amplification cycles required to reach the threshold value for both universal and specific targets.

## Data Availability

All program scripts and data are available at the open-access repository Zenodo: https://doi.org/10.5281/zenodo.17141784.

## SUPPLEMENTAL MATERIAL

Captions mbio.03566-24-s0001.docx Captions for Tables S1 to S4.

Table S1 mbio.03566-24-s0002.xlsx Cellular component analysis for the top 100 scored genes.

Table S2 mbio.03566-24-s0003.xlsx Biological process analysis for the top 100 scored genes.

Table S3 mbio.03566-24-s0004.xls CHI, DBI, and PDS for all genes.

Table S4 mbio.03566-24-s0005.xls CHI, DBI, and PDS for genes of interest.

ASM does not own the copyrights to Supplemental Material that may be linked to, or accessed through, an article. The authors have granted ASM a non-exclusive, world-wide license to publish the Supplemental Material files. Please contact the corresponding author directly for reuse.
