# Supplementary material for: A phylogeny-based method in detecting the species-specialized genes in microbes and its application to a marine symbiont bacterium
Source: mBio. 2025 Oct 29;16(12):e03566-24. doi: 10.1128/mbio.03566-24 (PMC12691675; doi:10.1128/mbio.03566-24)
Supplement: Captions — for Tables S1 to S4. [file mbio.03566-24-s0001.docx]

**A phylogeny-based method in detecting the species-specialized genes in microbes and its application to a marine symbiont bacterium**

**SUPPLEMENTARY MATERIAL**

**Supplemental Table Legends**

**Table S1.** Cellular Component analysis for the top 100 scored genes. This table presents the results of a Gene Ontology (GO) cellular component enrichment analysis for the 100 genes with the highest Phylogeny Deviation (PD) scores identified in *Candidatus Endobryopsis kahalalidifaciens* (cEK). See Methods for details.

**Table S2.** Biological Process analysis for the top 100 scored genes. This table presents the result of a Gene Ontology (GO) biological process enrichment analysis for the 100 genes with the highest PD scores in cEK. See Methods for details.

**Table S3.** CHI, DBI, and PDS for all genes. This table compiles the Cluster Homogeneity Index (CHI), Distance-Based Individuality (DBI), and Phylogeny Deviation Score (PDS) for all genes analyzed across the cEK genome and 196 related Flavobacteriaceae species. CHI measures cluster uniformity, DBI assesses sequence individuality, and PDS quantifies deviation from phylogenetic expectations.

**Table S4**. CHI, DBI, and PDS for genes of interest. This table focuses on the CHI, DBI, and PDS values for key recombination-related genes (ruvA, ruvB, ruvC, recG, ligA) identified in cEK

**Notes:**

- All data and scripts are available at DOI: <https://doi.org/10.5281/zenodo.17141784>.
- Tables are in Excel format and can be generated by scripts in the address above.
